# Medications, epilepsy and climate change: Added layers of complexity

**DOI:** 10.1002/bcp.70108

**Published:** 2025-05-27

**Authors:** Medine I. Gulcebi, Seyhmus Gavas, Sanjay M. Sisodiya

**Affiliations:** ^1^ Department of Clinical and Experimental Epilepsy UCL Queen Square Institute of Neurology London UK; ^2^ Chalfont Centre for Epilepsy Chalfont St Peter UK; ^3^ Department of Medical Pharmacology Marmara University School of Medicine Istanbul Turkey; ^4^ Epilepsy Research and Implementation Centre (EPAM), Marmara University Istanbul Turkey

**Keywords:** adverse weather events, global warming, neuropsychiatric drugs, pharmacokinetics

## Abstract

Climate change—the global crisis with pervasive health impacts—has adverse consequences for people with epilepsy (PWE) who have low quality of life due to poor seizure control, socioeconomic disadvantages and comorbidities. This review focuses on the potential effects of climate change on the pharmacological characteristics of antiseizure medications (ASMs), antipsychotics and antidepressants. We note that findings particularly obtained from physicochemical stability studies have been demonstrated experimentally for some specific environmental conditions whereas studies for clinical outcome effects are very limited. Carbamazepine, valproate, phenytoin or lorazepam appear to be ASMs at risk of being affected by high temperature and/or humidity. Even the stability of blood samples needs to be considered during transportation to therapeutic drug monitoring units, particularly for the PWE living in low‐income countries that are facing the most challenges of climate change effects attributed to low infrastructure and healthcare system capacity. We need more urgent research investigating drug responses of PWE regarding especially the effects of adverse weather events such as heatwaves on physicochemical stability or pharmacokinetics of drugs in a complex interaction with the vulnerabilities of individuals, accompanying neuropsychiatric disorders and geographical challenges. Then we will be able to develop pharmacological treatment strategies to improve the quality of life of PWE during adverse weather events.

## 1 INTRODUCTION

The need for action against the pervasive health effects of climate change is increasing as adverse weather events and temperature fluctuations became more frequent and severe.^1^, ^2^, ^3^, ^4^ Greenhouse gas (GHG) emissions are causing temperatures to rise, and rising temperatures are causing climate change, with its associated features such as more frequent and intense heatwaves,^5^, ^6^ as well as more extreme unseasonal temperatures short of heatwaves, and increased precipitation short of floods. This warming also increases the likelihood of extreme seasonal temperatures and intense rainfall. Disruption of health systems during adverse weather events, such as heatwaves, hurricanes, typhoons or wildfires, has serious adverse outcomes.^7^, ^8^, ^9^ Climate change has potential direct and indirect consequences for people with epilepsy (PWE) including deterioration of seizure control, aggravation of comorbidities and other consequences.^10^ Recently published studies have underlined the possible effects of climatic variables like temperature, atmospheric pressure, humidity or heatwaves on seizure frequency.^11^, ^12^, ^13^, ^14^ The increase in seizure frequency in PWE in Greece during summer months compared to winter months, for example, further highlights the added contribution of local geographical factors to temperature and other climatic fluctuations as a risk for precipitating seizures.^11^ Adverse weather events or global warming could play a significant role in triggering seizure precipitants or altering risk of epilepsy.^15^ There are both empirically demonstrated and plausible theoretical links between worsening manifestations of epilepsy and extreme weather.^16^, ^17^ Whilst the challenge is broadly described by the term ‘climate change’, its adverse effects are currently mainly mediated by proximate and acute phenomena, such as temperature extremes and adverse weather events.

**FIGURE 1:**
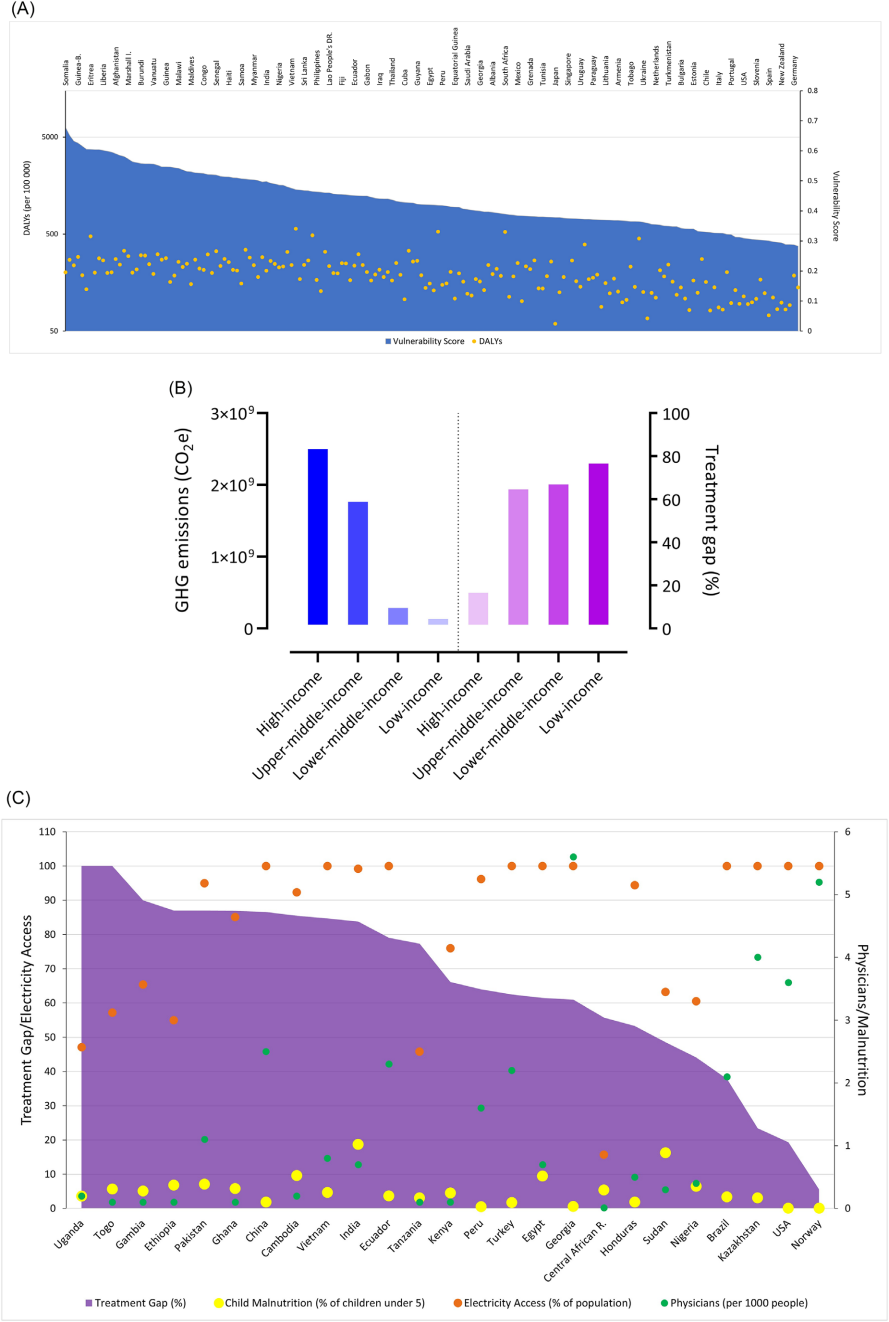
The factors regarding the importance of climate change for epilepsy. (A) Disability‐adjusted life years (DALYs) attributed to epilepsy and vulnerability of countries to climate change effects; the x‐axis shows countries (*n* = 180) for which vulnerability scores are available. Life‐supporting sectors; food, water, health, ecosystem services, human habitat and infrastructure with 36 Notre Dame‐Global Adaptation Index (ND_GAIN) indicators are used to measure vulnerability that the ND_GAIN uses to show the propensity of these countries to the negative impacts of climate change.^22^ Vulnerability scores for 2021, the most recently published scores by the ND‐GAIN, are shown. DALYs for epilepsy are shown, with the relevant scale on the right y‐axis, while vulnerability scores are on the left y‐axis. The counts for national distribution of age‐standardized DALYs for epilepsy were taken from the supplementary material of a recently published systematic review.^23^ The calculation of DALYs per 100 000 is based on the population of each country in 2016. All population data were taken from database.earth (https://database.earth/population/by‐country/2016 (accessed November 25, 2024). This website uses data sources from the United Nations. Countries with higher vulnerability scores to climate change than those countries with lower vulnerability scores tend to show higher DALYs. (B) Epilepsy treatment gap^24^ and GHG emissions by country income for 27 countries, obtained from Worldometer (https://www.worldometers.info/co2‐emissions/co2‐emissions‐by‐country/). (C) Epilepsy treatment gap^24^ showing the complex disposition of adaptive capacity with the existing treatment gap: climate change adds a further set of stresses and tensions to those already in existence. Primary epilepsy treatment gaps, which were described as the proportion of people with active epilepsy in study populations that are not receiving adequate or regular medical treatment at the time of the survey, were used. For countries with multiple study results, epilepsy treatment gap values were averaged.

Epilepsy affects around 50 million people of all ages worldwide. The burden of epilepsy is more than twice in low‐income countries compared to high‐income countries,^18^, ^19^ but average national GHG emissions are significantly lower in developing countries.^20^ Furthermore, epilepsy is one of the major contributors to global disease burden, as measured by the age‐standardized disability‐adjusted life years (DALYs) among neurological disorders,^18^, ^21^ especially in countries more vulnerable to climate change effects (Figure 1A). Countries with higher vulnerability scores to climate change are also those that tend to have higher values of DALYs. Climate change overall, therefore, will affect most those nations contributing least to its causation, nations that may already have the highest disease burden and largest treatment gap, for epilepsy (Figure 1B). In these countries, adaptive capacity indicators for medical staff, malnutrition or electricity also accompany the treatment gap (Figure 1C). The large gap in DALYs attributed to epilepsy between high‐income and low‐income countries has also been highlighted.^23^

**FIGURE 2:**
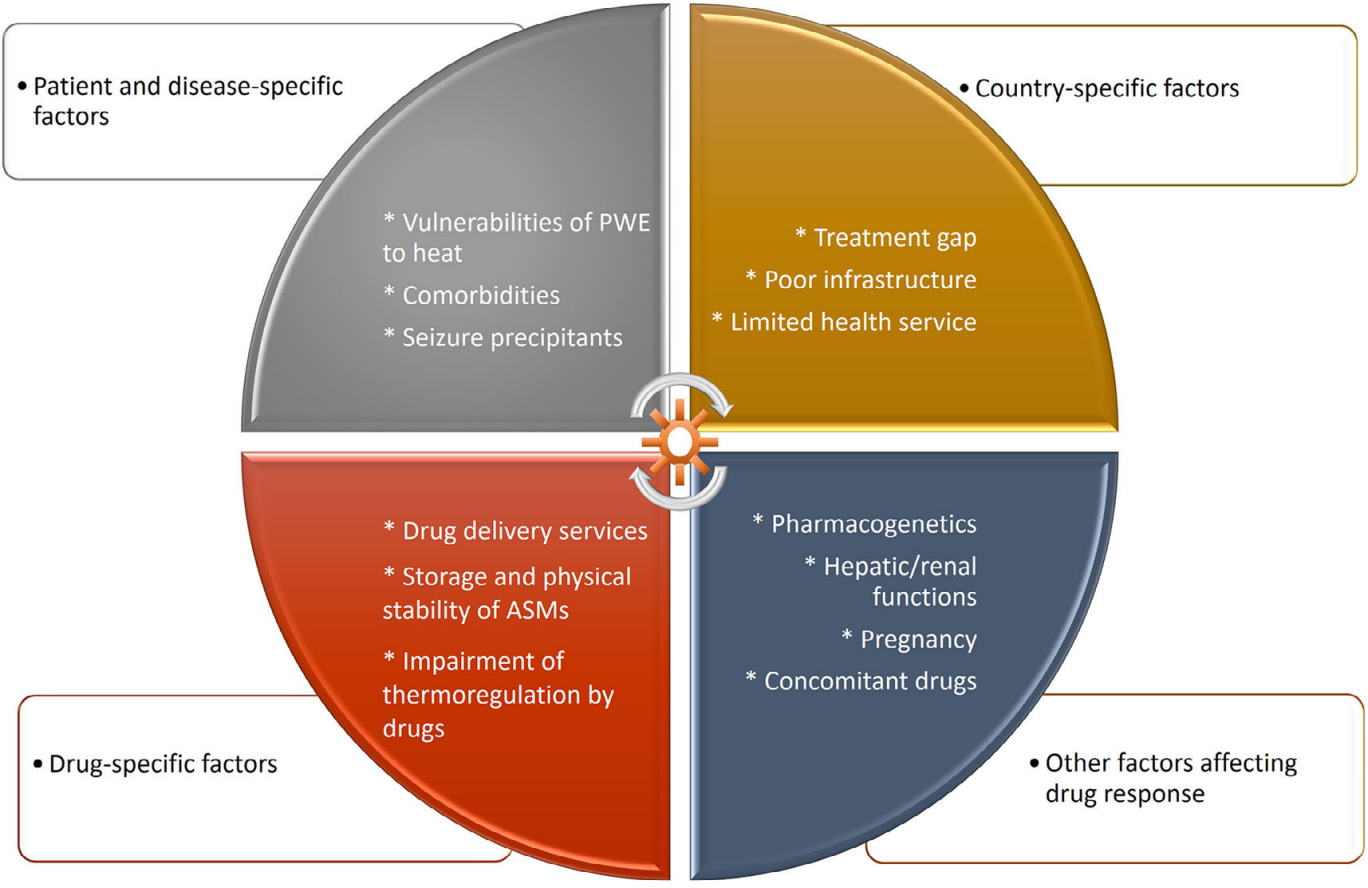
Multiple interacting factors contributing to the potential effects of adverse weather events on drug response of PWE. The vulnerability of PWE to climate change, disease‐specific factors, country‐specific factors and other factors affecting drug response might all be influenced by climate change.

Since quality of life for PWE is strongly associated with seizure control,^25^ the possibility of loss of seizure control due to climate change effects demands attention. Antiseizure medications (ASMs) are central to seizure control for most PWE. Adherence to, and therefore also supply of, ASMs is a key factor in treatment success.^26^ Up to 50% of PWE have comorbid conditions like depression, anxiety, dementia, migraine or heart disease, and the prevalence of most of these conditions is higher than the general population.^27^, ^28^, ^29^ Moreover, people with drug‐resistant epilepsy are often on polytherapy in addition to ASMs for the broad spectrum of comorbidities of epilepsy that greatly influence quality of life.^30^ An obvious impact of adverse weather events is through disruption of supply chains,^31^ potentially leading to compromised seizure control. Difficulties accessing ASMs, and the resulting adverse health outcomes, have already been reported related to the winter storm in the US in 2021, and (even if the earthquake is a natural disaster rather than an adverse weather event), the 2011 earthquake in Japan: both are pertinent examples indicating the risks of supply chain disruption.^32^, ^33^ Adverse weather events may affect the benefits of ASMs in other ways (Figure 2); for example, the pharmacological characteristics of ASMs may be affected by climate change, primarily through extreme temperature and humidity levels. For many drugs, including many ASMs, a strong relationship exists between the administered dose, serum level and pharmacodynamic effects at the sites of action.^34^, ^35^ For an optimum drug response, drugs usually need to reach to their minimum effective concentrations in blood and ideally levels should be maintained within a therapeutic range.^36^ Intra‐ and inter‐individual variability in drug response is affected by well‐known endogenous and exogenous factors, such as genetic factors and circadian and seasonal variations. ASMs maybe considered at risk to the impact of climate change since many have narrow therapeutic windows: even slight variations in their pharmacokinetics could lead to therapeutic failure or unwanted effects, for instance as can occur following switching between ASM brands.^37^, ^38^, ^39^, ^40^ This review aims to highlight the potential effects of seasonality and climate change, with its associated adverse weather events, on the pharmacological characteristics of medications commonly used in epilepsy, including ASMs, antipsychotics and antidepressants. The review brings out actions that can, and may need to, be taken even now and highlights the gaps in knowledge yet to be addressed.

## 2 EFFECTS OF HIGH TEMPERATURE AND/OR HUMIDITY ON THE PHYSICOCHEMICAL STABILITY OF ASMS

Therapeutic failure^41^ or adverse drug reactions^42^ can result from impairment of the physicochemical stability of ASMs or alteration of their pharmacodynamics and pharmacokinetics with extreme temperature and/or humidity exposure (Figure 2). The stability of drugs, defined as the ability of a pharmaceutical formulation of a drug or a drug product to retain the intended chemical, physical or microbiological properties during the time of its storage and use,^43^ is important in determining storage conditions and shelf‐life, and underpins the quality, safety and efficacy of drug products.^44^ The stability of a drug can be evaluated along two dimensions: physical stability and chemical stability. Physical stability is directly involved in drug release. Following oral intake of a drug, disintegration of the formulation and subsequent dissolution of drug molecules in the gastrointestinal fluid determine the absorption profile of the drug through cell membranes. Chemical stability determines the concentration of active pharmaceutical ingredients susceptible to chemical reactions, like hydrolysis or oxidation. The acceptable range for the actual amount of bioactive medication to be deemed chemically stable is within 90%–110% of the specified dose declared on the label of the drug product.^45^, ^46^ Physical instability refers to physical degradation of the drug formulation that could change the physical nature of the drug product and may affect drug release and bioavailability, whereas chemical instability refers to chemical degradation that could impact the intrinsic property, the chemical structure of the drug molecule, which could reduce drug potency or pose a safety risk.^47^

Storage conditions, including indoor and outdoor temperatures, can alter drug stability.^48^ Specific storage temperatures for ASMs generally range between 15 and 30 °C, with exceptions for some liquid formulations, such as gabapentin or lorazepam, which require refrigeration (Supplementary Table S1).^49^, ^50^ There is limited information on the influence of ambient temperature, humidity or storage conditions on distinct drug formulations of ASMs. Decreased in vitro dissolution rates for tablets of phenytoin, carbamazepine or sodium valproate can result from exposure to high temperature and/or humidity during storage (Table 1).^51^, ^52^, ^58^, ^59^, ^61^ Active ingredient analysis for carbamazepine and phenytoin tablets that were marketed in Vietnam showed that only 35% of tablets contained the amount of drug stated on the box, which has been attributed to different sensitivities of ASMs to different storage conditions (variations in light, humidity and temperature).^63^ Interestingly, dissolution rates of carbamazepine tablets from different manufacturers were affected by high temperature and/or humidity exposure to varying degrees.^51^, ^52^ The actual consequences of these effects have not yet been well studied, but we again refer to regulatory authority recognition of the risks of switching brands^64^ as a surrogate for potential adverse outcomes resulting from altered drug availability due to compromised physical stability. Moreover, it must be noted that the weather events that may have compromised drug stability may also at the same time increase risk of seizure occurrence through additional contemporaneous routes, such as sleep deprivation,^10^ which would not be an issue in general when drug bioavailability concerns relate to brand switching alone. Disentangling the multiple factors that might be relevant for PWE around adverse weather events will be challenging. The effect of storage conditions on the physicochemical stabilities of liquid formulations of some ASMs has also been examined (Table 1).^50^, ^53^, ^57^, ^62^ For instance, the Food and Drug Administration (FDA) recommends storing oral solutions of gabapentin under refrigeration (Supplementary Table S1); however, extemporaneously prepared oral suspensions of gabapentin showed crystallization at 5 °C for gabapentin oral suspensions prepared from capsules or bulk drug powder following storage in a refrigerator for 1 day or 7 days, respectively.^49^ According to household surveys, the kitchen, bathroom, living room or bedroom are the main storage areas in the home for drugs where exposure to sudden humidity and temperature is possible.^65^

**TABLE 1:** Examples of the effects of storage conditions on stability of ASMs, antipsychotics and antidepressants.

Drug name	Pharmaceutical formulation	Storage conditions	Exposure duration	Degradation	Reference
Carbamazepine	Oral tablets repackaged in bottles and oral solution	Oral tablets (Tegretol and Tegral dispensed in strip seals) of carbamazepine stored at 40 °C, 50 °C or 60 °C for 6 months, 3 months and 1 month respectively, at 75% relative humidity. Oral tablets removed from strips, placed in bottles, and exposed daily to 40 °C/97% relative humidity for 5 min per day for 1 month. Oral tablets (Finlepsin, dispensed in bottles) exposed to 25 °C or 40 °C/97% relative humidity for 5 min per day for 1 month by removing bottle caps daily.	1 month, 3 months or 6 months	Tegretol tablets were not affected. Tegral tablets showed increased dissolution and disintegration at 50 and 60 °C. Tablets that had been stored for 6 months at 40 °C/75% relative humidity or for 1 month at 40 °C/97% relative humidity were hardened and showed decreased dissolution compared to fresh Tegral tablets. For Finlepsin tablets, dissolution decreased at 25 °C/97% relative humidity and 40 °C/97% relative humidity. This effect was more profound at higher temperature (40 °C).	^51^
Carbamazepine	Tablet	40 °C/97% relative humidity Saturated water vapour at room temperature	6–7 days	Dissolution rates of some generics significantly decreased following exposure to 40 °C at 97% relative humidity for 6–7 days.	^52^
Diazepam	Injectable dosage form in ampoules	Ambient temperature of Mediterranean Summer temperature (could be over 40 °C)	10 years	No degradation	^53^
Diazepam, lorazepam	Injectable solution	Ambient temperature (15–30 °C), refrigerator (4–10 °C) or oven heated (37 °C)	Up to 210 days	Diazepam concentration reduced: 7% for refrigerated, 15% at ambient temperature, 25% at 37 °C. Lorazepam concentration reduced: 10% at ambient temperature, 75% at 37 °C.	^50^
Fluoxetine	Stock solutions	Ambient temperature (not specified)	30 days	40% decrease in fluoxetine concentration. No change for sertraline, venlafaxine or paroxetine.	^54^
Gabapentin	Extemporaneously prepared oral suspension	25 °C ± 2 °C/60% ± 5% relative humidity or refrigerator (5 °C)	0, 7, 14, 30, 45, 60, 75 and 90 days	Crystallization observed at 5 °C. All preparations remained stable at 25 °C ± 2 °C/60% ± 5% relative humidity for 90 days.	^49^
Lamotrigine	Tablets, repackaged	25 °C/60% relative humidity	6 months	No degradation	^55^
Levetiracetam	Oral solution, drawn into 1 mL and 10 mL amber polypropylene oral syringes.	Room temperature (20–25 °C) or refrigerator (2–8 °C)	Up to 6 months	At least 97% of the initial levetiracetam concentration was retained in all samples of 1 mL and 10 mL oral syringes at both refrigeration and room temperatures.	^56^
Lorazepam	Injectable dosage form, repackaged	Spring–summer months 9.2 °C to 35.9 °C, mean monthly ambient temperature	60 days	Temperature‐dependent degradation in concentration for lorazepam. No change for midazolam.	^57^
Olanzapine	Stock solution	Ambient temperature (not specified)	1 year	Significantly decreased concentration for olanzapine. No change for clozapine, haloperidol, or risperidone concentrations.	^54^
Phenytoin	Extended‐release oral capsules of phenytoin	30 °C/65% relative humidity or 40 °C/75% relative humidity	4 weeks	Dissolution decreased from 77.56 ± 3.83% to 42.34 ± 4.31%, and 63.96 ± 6.12 to 46.53 ± 2.91 in physical mixture and water granulated formulations, respectively, after 4 weeks exposure at 40 °C/75% relative humidity.	^58^
Phenytoin	Capsule	45 °C/85% relative humidity	7 days	50% decrease in dissolution rate	^59^
Sodium valproate	Enteric coated tablets, repackaged	Controlled room temperature, (25 ± 2 °C), accelerated (40 ± 2 C; 7 ± 1.5% relative humidity) and refrigerated (5 ± 3 C) conditions	4 weeks	Chemical stability of valproate was maintained for 28 days for all storage conditions. After 8 days of storage at accelerated conditions, tablets showed a 62% decrease in hardness.	^60^
Sodium valproate	Tablets, repackaged	Room temperature (25 °C), 40 °C/75% relative humidity or refrigerator (2–8 °C)	3, 10, 21 or 49 days	Significant decrease in the dissolution rate of tablets. No change in concentration.	^61^
Topiramate	Oral solution	Room temperature (20 °C to 25 °C) or refrigerator (2 °C to 8 °C)	90 days	No degradation.	^62^

Forced degradation studies examine the effects of various stresses, such as high temperature, humidity or acidic and alkaline pH conditions, on drug stability for distinct pharmaceutical formulations of drugs under controlled conditions.^44^ According to the climatic zone categorization of the “International Council for Harmonisation of Technical Requirements for Pharmaceuticals for Human Use (ICH) along with WHO”, there are four different zones representing the climates of various geographic regions to guide the pharmaceutical industry for drug stability testing.^66^ For example, climatic zone 1 identifies temperate regions such as the USA or UK, where the storage conditions should be 21 °C with 45% relative humidity.^67^ On the other hand, for climatic zone 4, which includes countries considered hot and humid, such as Brazil or Singapore, the required storage conditions are 30 °C with 75% relative humidity. Airtight and light‐resistant containers should be used for storage of light‐sensitive drugs to sustain drug stability.^68^, ^69^ Among oral formulations of ASMs, carbamazepine (tablet, chewable; tablet, extended‐release; oral suspension), felbamate (tablet), lamotrigine (tablet), levetiracetam (tablet; oral solution), oxcarbazepine (oral suspension), phenobarbital (tablet; oral suspension), phenytoin (capsule; oral suspension), topiramate (capsule; extended‐release), sodium valproate (capsule; oral suspension) and zonisamide (capsule) are listed as drugs that the manufacturer recommends protecting from light (Supplementary Table S1).^70^ An important general complication causing reduced bioavailability of drugs is repackaging: the removal of tablets from their original packaging and placement in other storage.^71^ Dose administration aids (DAAs) have been in use for more than three decades and are intended to improve adherence in chronic drug treatment, but DAA use may compromise stability of drugs in tropical or desert regions that are characterized by increased heat or humidity or sunlight compared to temperate regions.^72^ The types of DAAs or storage conditions are also relevant.^73^ For example, despite a long shelf life, the lack of recommendations for special storage conditions for phenytoin mean that inadvertent exposure to high temperature and humidity decreased the bioavailability of repackaged phenytoin capsules, resulting in breakthrough seizures in one patient report (Table 2).^59^ Absorption of repackaged immediate release or enteric‐coated sodium valproate tablets fell due to incomplete dissolution of drug molecules following exposure to high humidity.^60^, ^61^ No clinical consequences have yet been reported for this observation, though even slight alteration in valproate pharmacokinetics following substitution with generic formulations has been reported to provoke seizures.^80^ Although high temperature and/or humidity were not investigated, repackaging of lamotrigine tablets or oral solution of levetiracetam had no effect on their stabilities at room temperature (Table 1).^55^, ^56^

**TABLE 2:** Case reports of potential adverse interactions between effects of adverse weather events and various neuropsychiatric medications.

Drugs	Age and sex	Other drugs	Country	Adverse weather effects	Clinical outcome	Clinical pharmacology outcome	Reference
**Carbamazepine**	28‐year‐old man	Sodium valproate	US	Stability: Damp carbamazepine tablets Up to 65% reduction in dissolution due to high range of humidity exposure in the rainstorm.	Status epilepticus	Lower carbamazepine concentration compared to previous levels. Sodium valproate concentration was within therapeutic ranges.	^41^
**Clozapine**	60‐year‐old man	Sodium valproate	Brazil	Adverse drug reaction: Heatstroke, collapsed on the floor, with a raised temperature of 41.9 °C on the seventh day of a 27‐day heatwave in January and February of 2014, 39 °C air temperature.	Heatstroke Clozapine was reintroduced but on the 4th day represented with hyperthermia (38–38.9 °C).	Clozapine dose was reduced to 400 mg/day from 600 mg/day to avoid heat stress effect.	^74^
**Clozapine**	32‐year‐old man	None	UK	Adverse drug reaction: Heatstroke, working as a builder's labourer, found unconscious and seizing on an open building site with a raised temperature of 42.9 °C in the middle of a 3‐day heatwave, 26 °C air temperature.	Heatstroke A long history of alcohol, glue, cocaine and cannabis abuse; at the time of admission, they were unquantified. Liver failure, rhabdomyolysis, adult respiratory distress syndrome and disseminated intravascular coagulation.	An initial period of neuroleptic‐free assessment was decided to restart clozapine.	^75^
**Phenytoin**	31‐year‐old man	Phenobarbital	US	Stability: Dry and brittle phenytoin capsules with brown discoloration Repackaging the capsules into unheated and unairconditioned country cabin, possibly at 38–45 °C and 100% relative humidity.	Loss of seizure control Fresh phenytoin tablets used in a slightly increased dose.	Significantly lower phenytoin concentration compared to previous levels.	^59^
**Risperidone**	47‐year‐old man	Fluphenazine Benztropine Sodium valproate	US	Adverse drug reaction: Hyperthermia, was found unconscious on the street, with a raised body temperature of 41 °C in hot weather (about 32C).	Cerebrovascular accident following significant hyperthermia. Cerebellar damage was shown on brain MRI. He was treated with active cooling with ice packs and cool water bladder irrigation.	None	^76^
**Topiramate**	36‐year‐old man	None	Italy	Adverse drug reaction: Oligohydrosis and decreased sweat production following painting a house in a hot and sunny environment.	Heatstroke A long‐time history of alcohol abuse, supported by MRI findings.	None	^77^
**Topiramate**	40‐year‐old man	Sodium valproate	US	Adverse drug reaction: Heatstroke, found at home unresponsive and “very hot to touch” in July.	Heatstroke Death due to heatstroke and cardiac arrest, history of diabetes.	Serum topiramate and sodium valproate concentrations were within therapeutic ranges.	^78^
**Zonisamide**	24‐year‐old man	Sodium valproate, sodium bromide	Japan	Adverse drug reaction: Hyperthermia in Dravet syndrome patient.	Fever and hyperthermia‐induced seizures and status epilepticus.	Switching from zonisamide to perampanel improved seizure frequency, and status epilepticus no longer occurred.	^79^

An interesting and potentially important issue is the impact of climatic factors on the stability of ASMs and their metabolites in blood samples collected from the PWE for therapeutic drug monitoring (TDM).^81^ Although the clinical implications, if any, are unknown, a significant decrease in phenytoin concentration was detected following storage of blood samples in serum separator tubes at 32 °C or 37 °C for 1 day compared to refrigerated samples (Table 1).^82^, ^83^ Conversely carbamazepine, sodium valproate and phenobarbital were stable, with no change in their levels under the same storage conditions. Another study investigating the stability of carbamazepine and its active metabolite, carbamazepine epoxide, in plasma under various conditions of storage found that storing plasma samples from patients at unspecified room temperature for 1 month (as a proxy for having sometimes having to transport samples long distances) caused a significant reduction in the concentration of carbamazepine epoxide whereas carbamazepine concentration did not change.^84^ Since dosing for some ASMs may be adjusted with respect to their blood levels, such laboratory results might be misleading for clinicians, especially in regions where the samples have to travel long distances to reach the laboratory. For instance, among 12 countries in South America, only Brazil, Argentina, Colombia, Chile and Uruguay have TDM clinical units,^85^ which indicates that all the other countries need to send blood samples abroad for TDM analysis of ASMs when necessary. When sending serum samples to foreign laboratories, transport time should not be delayed by attempting to batch additional samples. Serum samples should be stored at 4–8 °C for a maximum 7 days to maintain the quality of the samples^86^, ^87^ in order to achieve accurate results for the individuals.

All these studies indicate the urgent need for further studies on the stability of ASMs in the context of current high ambient temperature and/or humidity fluctuations, at which level, and to what degree. There are modifying and confounding aspects such as brand types, different formulations or repackaging methods in addition to distinct study designs.^88^ More importantly, formal stability studies—especially those that can be considered historical—probably do not address the actual temperature and humidity profiles we are seeing now with increasing frequency and intensity, and mostly they do not investigate clinical consequences, except for a few cases as summarized in Table 2. Therefore, we need further studies that simulate current, real‐world adverse weather events and give information about taking preventive measures against degradation of drug stability. In addition, current storage condition stipulations could play an important role in the shelf life of the drugs: with longer advertised shelf life, there may be more risk of drugs being exposed to multiple adverse weather events.

## 3 DRUG RESPONSE TO ASMS: THE IMPACTS OF SEASONS AND ADVERSE WEATHER EVENTS

Pharmacokinetics may be affected by environmental conditions or body temperature.^89^, ^90^ We note again that climate change is affecting temperatures, with more extreme and unseasonal temperatures, heatwaves and precipitation. Few studies have investigated the role of seasonal variations on ASM pharmacokinetics and drug response.^42^, ^91^, ^92^, ^93^ A marked decrease was found in the serum phenytoin concentration on summer days compared to other seasons in PWE in Israel, a subtropical country, with a hot and humid summer climate^92^ and in PWE in Denmark, during the unusually hot summer days of 1976, without any dose changes.^93^ As phenytoin is present in sweat and is metabolized by the liver, extreme perspiration or change in the activity of hepatic cytochrome‐P450 (CYP) enzymes were suggested as underlying mechanisms for seasonally reduced phenytoin levels.^93^ Induction of CYP3A4 metabolism by high ultraviolet light intensity and high vitamin D levels may reduce the levels of immunosuppressant drugs such as tacrolimus and sirolimus, during sunny and hot summer months^94^, ^95^, ^96^: other substrates of CYP3A4, such as phenytoin, carbamazepine and some benzodiazepines (diazepam, midazolam, alprazolam and triazolam),^97^ might also be affected. PWE receiving monotherapy with carbamazepine or valproic acid had lower minimum (*C*_min_) and maximum concentrations (*C*_max_) in spring compared to autumn in Russia and these changes in levels were correlated with increased number and severity of seizures in spring.^91^ On the other hand, a simulation study which used published data to model pharmacokinetic changes during exercise stress or heat exposure concluded that in this situation hepatic clearance of drugs in general may decrease.^98^ Similarly, exposure to high ambient temperature has also been associated with additional decline of kidney function in individuals with chronic kidney disease.^99^ Thus, as the use of ASMs in individuals with hepatic or renal disease is already known to be complex,^100^, ^101^, ^102^ adverse weather events may be of importance for causing additional pharmacokinetic changes for ASMs, but this has yet to be demonstrated empirically.

Increased vulnerability of individuals to adverse drug reactions needs to be considered as a potential outcome of the weather manifestations of climate change. Heatwaves are associated with increased mortality and morbidity rates, for which age and drugs play key roles^103^, ^104^ and adverse drug reactions can develop more frequently during such events.^2^, ^105^ Heatwave‐related adverse drug reactions were investigated by Sommet et al.^2^ for two separate adverse weather events in France, in 2003 and 2006. The intense and short heatwave in 2003, which caused 14 800 additional deaths overall, was compared with the less intense but longer heatwave in 2006 (associated with 2000 additional deaths overall), for the frequency of serious adverse drug reactions and the culprit drugs. Although the frequency of adverse drug reactions did not change between the two heatwaves, the culprit drug groups were different. Cardiovascular drugs (digoxin, ACE inhibitors or beta receptor blockers), neuropsychiatric drugs (antidepressants, neuroleptics, antiparkinsonian drugs or ASMs), oral antidiabetics, proton pump inhibitors and oral anti‐infection drugs were associated with heatwave‐related adverse drug reactions, especially with metabolic, renal or neuropsychiatric outcomes, for both heatwaves. Diuretics, antiparkinsonian drugs and ASMs were more frequently involved in adverse drug reactions in the 2006 heatwave compared to 2003, whereas beta blockers and proton pump inhibitor‐related adverse effects were more prominent in 2003, which probably reflects better preparedness after the heatwave in 2003.^2^ Some ASMs, particularly topiramate and zonisamide, may predispose to hyperthermia or heatstroke in some PWE by disturbing thermoregulation via insufficient sweat production during high ambient temperatures (Table 2);^77^, ^78^, ^79^, ^106^, ^107^ the risk may be greater for paediatric patients and could be reduced by decreasing the topiramate dose (if possible), ensuring good hydration and providing local environmental cooling.^106^, ^107^ According to the Centers for Disease Control and Prevention (CDC), topiramate (by decreasing sweating), carbamazepine (through dizziness and weakness, especially with higher doses) and oxcarbazepine (by increased sweating and increased urination) could contribute to heat sensitivity.^108^ There are also supportive findings for the link between climatic factors and cutaneous adverse drug reactions induced by ASMs.^42^, ^109^, ^110^, ^111^ There was a marked increase in the incidence of phenytoin rash during summer compared to other seasons in people living in the USA.^42^ The highest rate for cutaneous reactions within 3 weeks of initiation of phenytoin therapy (20.6%, 13 of 63 individuals), was observed during June, July and August; there were no such reactions (in 79 individuals) for individuals in whom treatment was initiated in December, January and February; this difference was attributed to the protective effects of a more activated immune system in winter.^42^ It is unknown to what extent climate change has contributed to this seasonal effect. Further, there are case reports of photosensitivity associated with lamotrigine that occurred after individuals were exposed to sunlight or with solarium usage.^109^, ^110^, ^111^ The patients experienced adverse cutaneous reactions^109^, ^111^ including toxic epidermal necrolysis^110^ that resolved after the discontinuation of lamotrigine. Global warming and stratospheric ozone depletion may put individuals at higher risk of medication‐induced photosensitivity via exposure to higher levels of solar ultraviolet radiation,^112^ which has been considered a potential stimulating factor for delayed cutaneous reactions to lamotrigine therapy.^109^

## 4 GEOGRAPHY, DRUGS AND CLIMATE CHANGE

Climate change impacts will not be the same across the whole world. Given the overlap of adverse weather events with poor air conditioning and/or tropical and subtropical geographical regions that are more sensitive to the effects of climate change,^113^, ^114^, ^115^ it is important to consider geographical factors in climate risks for PWE. Both the incidence and prevalence of epilepsy are higher in lower‐middle‐income countries,^113^, ^114^, ^115^ which are located in tropical regions. There are significant challenges for epilepsy treatment in low‐income countries in terms of availability and access to newer ASMs, poor quality of ASMs (e.g., drugs with insufficient active ingredient, mislabelled or counterfeit packaging) and storage conditions (e.g., poor temperature and humidity control).^19^ Even when there is an opportunity to use TDM to inform dosing, inappropriate transport of blood samples over long distances for laboratory analysis without a cold chain weakens the success of epilepsy treatment.^82^, ^116^ The treatment gap in low‐ and middle‐income countries imperils appropriate and comprehensive treatment of PWE.^24^ Perhaps unsurprisingly, a higher treatment gap for epilepsy, arising from many factors such as drug supply and affordability, appears to correlate with lower national greenhouse gas emissions (Figure 1B). Moreover, in low‐ and lower‐middle‐income countries, TDM is typically either unavailable or restricted to first‐generation ASMs rather than newer ASMs.^117^ The most commonly used ASMs in Nigeria (lower‐middle income country), Pakistan (lower‐middle income country) and Republic of South Africa (upper‐middle income country),^118^ for example, are first‐generation ASMs such as sodium valproate, carbamazepine or phenobarbital^119^, ^120^, ^121^: as discussed above, these ASMs appear to be most vulnerable when exposed to extreme temperatures and/or humidity.

## 5 NEUROPSYCHIATRIC DISORDERS ASSOCIATED WITH EPILEPSY: THE IMPACTS OF CLIMATE CHANGE

Depression, anxiety, bipolar disorder, schizophrenia and neurodegenerative diseases such as dementia or Alzheimer's disease are common comorbidities of epilepsy^27^: use of antipsychotics or antidepressants is therefore common in PWE.^30^ Epilepsy and ASMs can both be associated with cognitive disadvantages such as mental slowness and attention deficit problems^122^ that may compromise the ability to cope with adverse weather events or understanding health alerts related to weather. Cognitive impairment itself, which is common in PWE, has been identified as a risk factor for heat‐related illness in adverse weather events.^123^ On the other hand, ASMs have indications for neuropsychiatric disorders in addition to epilepsy, for instance, carbamazepine, valproic acid or lamotrigine for bipolar disorder, carbamazepine and gabapentin for peripheral neuropathy, carbamazepine for trigeminal neuralgia, or topiramate for migraine (https://www.nhs.uk/medicines/). Gabapentin rates tenth among the 200 most frequently prescribed drugs in the US in 2022, with an estimated 40 141 486 prescriptions, lamotrigine is 58th with ~11 704 668 prescriptions, topiramate is 84th with 8 210 654 prescriptions, and valproic acid is 174th with 2 969 360 prescriptions.^124^ It is important to note recent restrictions on valproate use.^125^ Therefore, climatic factors that affect these ASMs in epilepsy may be relevant also to these other conditions.

The storage of stock solutions of olanzapine, an antipsychotic, or fluoxetine, a commonly used antidepressant, led to significant concentration loss under unspecified ambient temperature (Table 1).^54^ Individuals with pre‐existing mental health conditions, elderly people and those with neurological diseases or individuals taking antipsychotics are likely to be at higher risk for the effects of adverse weather events.^126^, ^127^, ^128^ In older people, there may be impaired thermoregulatory control, due to various factors such as age‐related reduced sweat production through altered dynamic cutaneous vasodilation following heat exposure or chronic cardiovascular diseases such as heart failure.^129^ Since sweating is a mechanism for reducing body temperature in extremely hot weather, reduced sweating may result in increased sensitivity to heat.^130^ Furthermore, antipsychotics (neuroleptics), antidepressants, anticholinergics or antihistamines, in addition to cardiovascular agents, may predispose people to heat‐related illness and heat‐related hospitalization, even in the absence of heatwaves, particularly during summer months, by disturbing autonomic responses or thermoregulatory control.^127^, ^131^, ^132^ An alteration of body temperature was reported for some individuals being treated with antipsychotics such as clozapine, olanzapine, risperidone or haloperidol (Table 2).^74^, ^75^, ^133^, ^134^ Case reports for a commonly used antipsychotic, risperidone, and the combination therapy of the tricyclic antidepressant, amitriptyline, with perphenazine, documented hyperthermia, resulting in a cerebellar syndrome in some individuals with schizophrenia.^76^, ^135^ The antimuscarinic drug group, used for treatment of excessive salivary drooling in some PWE (often with concomitant intellectual disability), may also increase the risk of heat intolerance by reducing sweating.^130^ Hyoscine and scopolamine, especially when combined with antipsychotics or carbonic anhydrase inhibitors (topiramate, zonisamide), may increase this risk.^136^ For hyoscine hydrobromide patches, a drug safety update reported a risk of a serious anticholinergic syndrome especially in children and elderly individuals when used off‐licence; for instance, applying the patches more than one at a time or continuously without a break or for long term.^137^ A recently published meta‐analysis reported that drugs with strong antimuscarinic properties, such as atropine, may increase body temperature at ambient temperature ≥30 °C.^138^ Although there are no published case reports for the clinical importance of using antimuscarinic drugs in PWE already taking antimuscharinic drugs, their use may lead to heat intolerance if they are exposed to high ambient temperatures.^139^, ^140^, ^141^

To understand these risks better, it is important to establish the mechanisms involved for different drug groups, such as whether they disturb thermoregulation via disturbed heat production, heat conservation or heat loss. For example, topiramate and zonisamide are both carbonic anhydrase inhibitors and dysfunction of sweat production was suggested as the mechanism,^142^ whereas Moore et al.^143^ underlined the systemic effect of dehydration caused by carbonic anhydrase inhibitors as they found no change in the sweat rate following direct inhibition of carbonic anhydrase in healthy people following exercise in heat. Nevertheless, we need to consider the physiological differences between PWE and healthy people while evaluating these findings. Antipsychotics, selective serotonin reuptake inhibitors (SSRIs) and some ASMs like carbamazepine and valproate may decrease thirst sensation and thereby could make people more vulnerable to development of heat‐shock and heat stroke.^3^

TDM could be helpful for dose adjustment in individuals treated with antipsychotics.^144^ The concentration of the atypical antipsychotics—aripiprazole, quetiapine, clozapine, risperidone and olanzapine—in whole blood or serum samples decreased significantly when they were exposed to ambient temperature for 2 days instead of being stored in a refrigerator.^144^ Lithium concentrations were 25% higher for an unchanged dose during the summer months in India, a subtropical region, compared to October (used to represent the mean temperature of the year),^145^ with similar findings reported from the US and Italy.^146^, ^147^ Variability in the serum concentration of lithium in people with bipolar disease over different seasons^145^, ^146^, ^147^ shows a need for close monitoring of its level in the context of climate variability including climate change.

## 6 SYNDROMES, DRUGS AND CLIMATE CHANGE

Despite the lack of high‐quality evidence for climate variability (including climate change) effects on drug response, it should be noted that PWE have reported temperature and/or seasonality as a triggering factor for their seizures.^15^ Individual‐ or disease‐specific vulnerabilities should be taken into account in order to estimate the impact of adverse weather events on drug response in PWE. Seizure‐triggering factors such as fever, poor sleep, fatigue and stress may be exacerbated during adverse weather events. For example, sleep cycle disruption is an important seizure precipitant that has been reported after adverse weather events, particularly in older people or low‐income populations.^148^ The adverse effects of ASMs associated with hyperthermia may be particularly important in people with temperature‐sensitive seizures, such as those seen in Dravet syndrome. Furthermore, climate anxiety may affect individuals with any type of epilepsy syndrome. Additional intellectual difficulties may further compromise care as PWE may not be able to communicate their thermal discomfort.

## 7 DISCUSSION

Documentation of the effects of adverse weather events on pharmacological characteristics of ASMs, antipsychotics or antidepressants, and the resulting clinical consequences, is very scant and often published some time ago when climate change—and accompanying global average surface temperatures—may have been lower, and when heatwaves may have been less frequent and less intense. We therefore need more research urgently, especially to investigate the effect of extreme temperatures on drug response in PWE. Such efforts will require the development of more sophisticated experimental models and transdisciplinary research. Whilst we attempt to draw out risks that may exist, we acknowledge that some inferences are indirect. We note some reported findings have been shown empirically (especially physicochemical stability studies), while some proposed effects (clinical consequences effects) have been studied little. The existing information in the literature indicates the need for evaluation of the potential effects of climatic variables on ASMs and other drugs commonly used in PWE both before drug intake (change in physicochemical stability) and after drug intake (change in pharmacokinetics or stability of samples used for TDM).

Within the past 170 years, global temperatures have increased by approximately 1.1 °C, an unprecedented change over thousands of years of human history.^149^ Such rapid warming has greatly increased the frequency, intensity and duration of extreme heat events, such as heatwaves, since the 1950s.^150^ According to the US Environmental Protection Agency (US EPA), the frequency of heatwaves in major cities of the USA has increased from an average of two heatwaves per year during the 1960s to six per year during the 2010s and 2020s.^151^ The European State of the Climate (ESOTC) has reported that 23 of the 30 most severe heatwaves in Europe since 1950 occurred in the last 20 years.^152^ Future projections suggest that extreme heat will intensify significantly in most land regions by the end of the century, with increases in the frequency, duration and severity of hot days and nights.^5^ Unless significant steps are taken to mitigate climate change, these extremes are projected to continue to intensify throughout the twenty‐first century.^149^

The relationship between extreme temperatures and seizure control appears to be complex. Reports suggest high, or unseasonal, ambient temperature leads to more frequent seizures.^12^, ^128^, ^153^ The underlying mechanisms are not well understood and we do not currently know if and how ASM‐related issues may be contributing, but this is worth investigating as adaptation to ASM‐related effects driven by climate change may be relatively simple. Of the five patients with a higher number of seizures during a heatwave period compared to their seizure frequency during a non‐heatwave period, all were on their full usual dose of ASMs, and three were using topiramate or zonisamide.^12^ Patient‐ and disease‐specific factors may play important roles in the development of consequences of adverse weather events in PWE under ASM therapy. PWE may be additionally vulnerable to climate change impacts due to underlying comorbidities such as sleep disturbance or affective disorder and due to physiological factors affecting pharmacokinetics such as age, hepatic/renal function, pregnancy, pharmacogenetics and co‐medications; understanding these factors could also lead to better understanding adaptation strategies. These pharmacological questions need to be addressed with well‐designed experimental and clinical studies. The issues are complex: for example, impairment of the physicochemical stability of ASMs before drug intake due to an adverse weather event might overlap with stability problems of blood samples collected for measurement of drug levels in a distant laboratory.

Research in low‐ and‐middle‐income countries facing additional challenges, such as the treatment gap for epilepsy, compromised infrastructure or limited health service for PWE, is particularly scarce and many such countries are among the most vulnerable as nations to the impacts of climate change. Most studies on climate impacts on neurological diseases have been conducted in high‐income countries.^128^, ^154^, ^155^ For instance, generic drugs are commonly used in lower‐middle‐income countries^119^ and frequent generic substitution of ASMs is a known risk factor for worsening of seizures or developing of adverse drug reactions, mainly caused by slight changes in the pharmacokinetics of these drugs despite the stated bioequivalence range (80%–125%) provided for these generics.^156^ There is an urgent requirement for more evidence from these countries, and research in these regions should be supported.

The fact that the impact of climate change on brain health is a relatively new area of research, the different vulnerabilities of countries, low reporting rates of adverse outcomes and the inability to fully understand how climate change consequences might affect the stability or effectiveness of drugs used by PWE limit what can be concluded and recommended. We emphasize the need to develop adaptation strategies for ASMs, whether used for epilepsy or other indications, as climate change will increase the frequency and severity of heatwaves.^157^ Testing conditions used in older studies may not cover the types and severity of environmental challenges we are seeing currently, as temperature and rainfall records, for example, are being so regularly broken across the world. Protocols should be created that allow rapid repeat testing as environmental conditions change. The physicochemical stability of ASMs, antipsychotics and antidepressants require careful consideration during extreme temperatures. Especially for PWE living in areas of high humidity, it may be advisable to not use DAAs, or to store their drugs in the refrigerator (if available) during hot and humid periods.^60^, ^61^ If a medication requires storage with a narrow temperature range, storage in the kitchen, bathroom or garage is probably inappropriate.^158^ For primary care and community pharmacies, storage in drug cupboards, or even car boots for emergency use on home visits, could cause exposure of drugs to temperature above 25 °C during hot days.^159^ Therefore, it would be important to educate individuals, pharmacists and primary care physicians to ensure safe storage, as the development of more heat‐ and humidity‐stable drug formulations may prove more challenging. Using air‐conditioning or moving medications into a shaded location at home may help maintain the physicochemical stability. In some circumstances, for example with anticipated floods or heatwaves that may compromise supply chains, stockpiling under proper storage conditions may be advisable. The mechanism underlying association of worsening of clinical measures with adverse weather events may in some cases be at least partly due to climate impacts on drugs, and this possibility merits investigation, as adaptation may be relatively straightforward and could provide greater individual security against the risks of climate change impacts. In any case, the education of healthcare professionals, supply chain operators and PWE themselves about potential risks to medications related to climate change impacts should be considered as another relatively easy adaptation. As one example, drug safety measures could be added to individual rescue protocols that many PWE already have.

Given the growing impact of climate change on global environments, it is crucial to conduct further research to understand how factors like temperature and humidity fluctuations, both seasonal and related to the effects of climate change, could consequentially alter the pharmacodynamics and pharmacokinetics of various drugs. Understanding, how temperature fluctuations may affect drug response is vital to ensure the safety, efficacy and proper dosing of medications in the context of climate change‐related stressors.

Research gaps that could be considered for drug response in a changing climate include:

- physicochemical stability of drugs,
- altered liver enzyme activity and other key factors in drug metabolism,
- adverse effects associated with disrupted thermoregulation,
- dysfunction in cellular components and networks,
- changes in expression and function of temperature‐sensitive proteins such as ion channels,
- vulnerable groups, such as the elderly and children,
- impacts of comorbidities and co‐medications.

Some of the actionable shorter‐term practical tips that may address the impacts of climate change on drug response include:

- incorporating monitoring of response to ASMs into clinical trials during heatwaves,
- developing clear guidelines for healthcare professionals on how adverse weather events might affect drug responses in PWE,
- reporting adverse drug reactions that may be linked to climate change‐induced environmental factors, especially in areas with extreme weather patterns,
- advice on safer storage of medications during extreme temperatures,
- integrating climate considerations into prescription practices

In conclusion, climate change, as a fundamental threat to our health,^160^, ^161^, ^162^ is posing an additional challenge for PWE requiring chronic pharmacological treatment, but one that may be relatively easily addressed, if the issues are actually thought about.

## AUTHOR CONTRIBUTIONS

MIG and SG prepared the figures. SMS wrote the original draft with contributions from MIG and SG. SMS conceptualized the study.

## CONFLICT OF INTEREST STATEMENT

The authors declare no conflicts of interest.

## Supporting information


**Table S1** Pharmaceutical formulations, recommended storage conditions and potential adverse drug effects concerning thermoregulation of ASMs, antipsychotics and antidepressants.

## Data Availability

Data available on request.
