# Supplementary material for: Medications, epilepsy and climate change: Added layers of complexity
Source: Br J Clin Pharmacol. 2025 May 27;91(8):2205–21. doi: 10.1002/bcp.70108 (PMC12304832; doi:10.1002/bcp.70108)
Supplement: Supplementary file 1 — Table S1 Pharmaceutical formulations, recommended storage conditions and potential adverse drug effects concerning thermoregulation of ASMs, antipsychotics and antidepressants. [file BCP-91-2205-s001.docx]

| Supplementary Table 1. Pharmaceutical formulations, recommended storage conditions and potential adverse drug effects about thermoregulation of ASMs, antipsychotics and antidepressants. | | | | | | |
| --- | --- | --- | --- | --- | --- | --- |
| **First generation ASMs** | Drug name | Formulation | Recommended storage temperature | Recommended storage humidity | Light-sensitivity | Adverse drug effects about thermoregulation |
|  | Carbamazepine | Oral tablets | < 30°C | Protect from moisture | Not specified | Fever and chills |
|  |  | Oral suspension | < 30°C | Not specified | Protect from light | Fever and chills |
|  |  | Solution for injection | 20°C - 25°C (Excursions permitted between 15°C to 30°C) | Not specified | Not specified | Fever |
|  | Ethosuximide | Oral capsules | 20°C - 25°C (Excursions permitted between 15°C to 30°C) | Not specified | Not specified | None |
|  |  | Oral suspension | < 30°C (Protect from freezing) | Not specified | Protect from light | None |
|  | Phenobarbital | Oral tablets | 20°C - 25°C | Protect from moisture | Protect from light | None |
|  |  | Solution for injection | 20°C - 25°C (Excursions permitted between 15°C to 30°C) | Not specified | Not specified | None |
|  | Phenytoin | Oral capsules | 20°C - 25°C | Protect from moisture | Protect from light | None |
|  |  | Oral suspension | 20°C - 25°C (Protect from freezing) | Not specified | Protect from light | None |
|  |  | Solution for injection | 2°C - 8°C (Store under refrigeration) | Not specified | Not specified | Sweating |
|  | Valproic acid | Oral capsules | 15°C - 25°C | Not specified | Not specified | Hypothermia, fever |
|  |  | Oral solution | < 30°C | Not specified | Not specified | Hypothermia, fever |
|  |  | Solution for injection | 15°C - 30°C | Not specified | Not specified | Hypothermia, fever |
| **Second or third generation ASMs** | Brivaracetam | Oral tablets | 25°C (Excursions permitted between 15°C to 30°C) | Not specified | Not specified | None |
|  |  | Oral solution | 25°C (Excursions permitted between 15°C to 30°C) | Not specified | Not specified | None |
|  | Cannabidiol | Oral solution | 20°C - 25°C (Excursions permitted between 15°C to 30°C) | Not specified | Not specified | Fever |
|  | Eslicarbazepine acetate | Oral tablets | 20°C - 25°C (Excursions permitted between 15°C to 30°C) | Not specified | Not specified | None |
|  | Everolimus | Oral tablets | 20°C - 25°C (Excursions permitted between 15°C to 30°C) | Protect from moisture | Protect from light | None |
|  |  | Oral solution | 20°C - 25°C (Excursions permitted between 15°C to 30°C) | Protect from moisture | Protect from light | None |
|  | Felbamate | Oral tablets | 20°C - 25°C | Not specified | Not specified | None |
|  |  | Oral suspension | 20°C - 25°C | Not specified | Not specified | None |
|  | Gabapentin | Oral tablets or capsules | 25°C (Excursions permitted between 15°C to 30°C) | Not specified | Not specified | Fever  (in patients 3 to 12 years of age) |
|  |  | Oral solution | 2°C - 8°C (Store under refrigeration) | Not specified | Not specified | Fever  (in patients 3 to 12 years of age) |
|  | Lacosamide | Oral tablets | 20°C - 25°C (Excursions permitted between 15°C to 30°C) | Not specified | Not specified | None |
|  |  | Oral solution | 20°C - 25°C (Excursions permitted between 15°C to 30°C, do not freeze) | Not specified | Not specified | None |
|  |  | Solution for injection | 20°C - 25°C (Excursions permitted between 15°C to 30°C, do not freeze) | Not specified | Not specified | None |
|  | Lamotrigine | Oral tablets | 25°C (Excursions permitted between 15°C to 30°C) | Store in a dry place | Protect from light | Fever, dry skin, sweating |
|  |  | Oral suspension | 25°C (Excursions permitted between 15°C to 30°C) | Store in a dry place | Not specified | Fever, dry skin, sweating |
|  | Levetiracetam | Oral tablets | 25°C (Excursions permitted between 15°C to 30°C) | Not specified | Not specified | None |
|  |  | Oral solution | 25°C (Excursions permitted between 15°C to 30°C) | Not specified | Not specified | None |
|  |  | Solution  for injection | 25°C (Excursions permitted between 15°C to 30°C) | Not specified | Not specified | None |
|  | Oxcarbazepine | Oral tablets | 20°C - 25°C (Excursions permitted between 15°C to 30°C) | Not specified | Not specified | Fever |
|  |  | Oral suspension | 20°C - 25°C (Excursions permitted between 15°C to 30°C) | Not specified | Not specified | Fever |
|  | Perampanel | Oral tablets | 20°C - 25°C (Excursions permitted between 15°C to 30°C) | Not specified | Not specified | None |
|  |  | Oral suspension | < 30°C (Do not freeze) | Not specified | Not specified | None |
|  | Pregabalin | Oral capsules | 25°C (Excursions permitted between 15°C to 30°C) | Not specified | Not specified | None |
|  |  | Oral solution | 25°C (Excursions permitted between 15°C to 30°C) | Not specified | Not specified | None |
|  | Rufinamide | Oral tablets | 25°C (Excursions permitted between 15°C to 30°C) | Not specified | Not specified | None |
|  |  | Oral suspension | 25°C (Excursions permitted between 15°C to 30°C) | Not specified | Not specified | None |
|  | Stiripentol | Oral capsules | 20°C - 25°C (Excursions permitted between 15°C to 30°C) | Store in a dry place | Protect from light | None |
|  |  | Oral suspension | 20°C - 25°C (Excursions permitted between 15°C to 30°C) | Store in a dry place | Protect from light | None |
|  | Tiagabine | Oral tablets | 20°C - 25°C | Protect from moisture | Protect from light | Dry skin, sweating |
|  | Topiramate | Oral capsules | <25°C | Protect from moisture | Not specified | Oligohidrosis and hyperthermia (especially in pediatric patients), hypothermia (with concomitant valproate use), fever |
|  |  | Oral tablets | 15°C - 30°C | Protect from moisture | Not specified | Oligohidrosis and hyperthermia (especially in pediatric patients), hypothermia (with concomitant valproate use), fever |
|  | Vigabatrin | Oral tablets | 20°C - 25°C | Not specified | Not specified | Fever |
|  |  | Oral solution | 20°C - 25°C | Not specified | Not specified | Fever |
|  | Zonisamide | Oral capsules | 25°C (Excursions permitted between 15°C to 30°C) | Store in a dry place | Protect from light | Oligohidrosis  and hypothermia |
| **Antipsychotics** | Aripiprazole | Oral tablets | 25°C (Excursions permitted between 15°C to 30°C) | Not specified | Not specified | None |
|  |  | Oral solution | 25°C (Excursions permitted between 15°C to 30°C) | Not specified | Not specified | None |
|  |  | Solution for injection | 25°C (Excursions permitted between 15°C to 30°C) | Not specified | Protect from light | None |
|  | Clozapine | Oral tablets | < 30°C | Not specified | Not specified | Sweating, fever |
|  |  | Oral solution | 25°C (Do not refrigerate or freeze) | Not specified | Protect from light | Sweating, fever |
|  | Haloperidol | Oral tablets | 20°C - 25°C | Not specified | Not specified | Hypothermia, hyperthermia |
|  |  | Solution for injection | 15°C - 30°C (Do not freeze) | Not specified | Protect from light | Hypothermia,  hyperthermia |
|  | Olanzapine | Oral tablets | 20°C - 25°C (Excursions permitted between 15°C to 30°C) | Protect from moisture | Protect from light | Dehydration |
|  |  | Powder for injection solution | 20°C - 25°C (Excursions permitted between 15°C to 30°C, do not freeze) | Not specified | Protect from light | Dehydration |
|  | Risperidone | Oral tablets | 15°C - 25°C | Protect from moisture | Protect from light | Hypothermia,  hyperthermia |
|  |  | Oral solution | 15°C - 25°C (Do not freeze) | Not specified | Protect from light | Hypothermia,  hyperthermia |
|  |  | Solution for injection | 2°C - 8°C (Store under refrigeration, If refrigeration is unavailable, it can be stored at temperatures not exceeding 25°C for no more than 7 days prior to administration. Do not expose unrefrigerated product to temperatures above 25°C) | Not specified | Protect from light | Hypothermia,  hyperthermia |
|  | Quetiapine | Oral tablets | 25°C (Excursions permitted between 15°C to 30°C) | Not specified | Not specified | None |
| **Antidepressants** | Citalopram | Oral tablets | 20°C - 25°C (Excursions permitted between 15°C to 30°C) | Not specified | Not specified | None |
|  |  | Oral solution | 25°C (Excursions permitted between 15°C to 30°C) | Not specified | Not specified | None |
|  | Duloxetine | Oral delayed-release capsules | 25°C (Excursions permitted between 15°C to 30°C) | Not specified | Not specified | Hyperhidrosis (in adults) |
|  | Escitalopram | Oral tablets | 20°C - 25°C (Excursions permitted between 15°C to 30°C) | Not specified | Not specified | Increased sweating |
|  |  | Oral solution | 20°C - 25°C (Excursions permitted between 15°C to 30°C) | Not specified | Not specified | Increased sweating |
|  | Fluvoxamine | Oral tablets | 20°C - 25°C (Excursions permitted between 15°C to 30°C) | Protect from high humidity | Not specified | Sweating |
|  | Fluoxetine | Oral tablets | 25°C (Excursions permitted between 15°C to 30°C) | Not specified | Not specified | Sweating |
|  |  | Oral capsules | 15°C - 30°C | Not specified | Protect from light | Sweating |
|  |  | Oral solution | 15°C - 30°C | Not specified | Protect from light | Sweating |
|  | Mirtazapine | Oral tablets | 20°C - 25°C (Excursions permitted between 15°C to 30°C) | Protect from moisture | Protect from light | None |
|  | Paroxetine | Oral tablets | 15°C - 30°C | Not specified | Not specified | Sweating |
|  |  | Oral suspension | ≤ 25°C | Not specified | Not specified | Sweating |
|  | Sertraline | Oral tablets | 20°C - 25°C (Excursions permitted between 15°C to 30°C) | Not specified | Not specified | Increased sweating |
|  |  | Oral solution | 20°C - 25°C (Excursions permitted between 15°C to 30°C) | Not specified | Not specified | Increased sweating |
|  | Venlafaxine | Oral extended-release capsules | 25°C (Excursions permitted between 15°C to 30°C) | Protect from moisture and humidity | Not specified | Sweating |
| ASMs: Antiseizure medications. All data regarding storage conditions and adverse drug effects have been taken from safety data sheets of particular drugs and formulations on FDA’s website. https://www.accessdata.fda.gov (accessed December 2024). | | | | | | |
|  |  |  |  |  |  |  |
